# X-ray phase-contrast tomography of cells manipulated with an optical stretcher

**DOI:** 10.1107/S1600577524003618

**Published:** 2024-06-11

**Authors:** Jan-Philipp Burchert, Jasper Frohn, Ulrike Rölleke, Hendrik Bruns, Boram Yu, Sophie-Charlotte Gleber, Roland Stange, Madleen Busse, Markus Osterhoff, Tim Salditt, Sarah Köster

**Affiliations:** a Institute for X-ray Physics University of Göttingen Friedrich-Hund-Platz 1 37077Göttingen Germany; b Cluster of Excellence Multiscale Bioimaging: from Molecular Machines to Networks of Excitable Cells (MBExC) University of Göttingen Germany; c RS Zelltechnik, 94508Schöllnach, Germany; d Biomedical Physics, School of Science Technical University Munich Boltzmannstraße 11 85748Garching Germany; e Munich Institute of Biomedical Engineering Technical University Munich Boltzmannstraße 11 85748Garching Germany; University of Malaga, Spain

**Keywords:** optical stretcher, X-ray phase-contrast tomography, high-resolution imaging, biological cells, X-ray stain

## Abstract

X-rays can penetrate deeply into biological cells and thus allow for examination of their internal structures with high spatial resolution. In this study, X-ray phase-contrast imaging and tomography is combined with an X-ray-compatible optical stretcher and microfluidic sample delivery. Using this setup, individual cells can be kept in suspension while they are examined with the X-ray beam at a synchrotron. From the recorded holograms, 2D phase shift images that are proportional to the projected local electron density of the investigated cell can be calculated. From the tomographic reconstruction of multiple such projections the 3D electron density can be obtained. The cells can thus be studied in a hydrated or even living state, thus avoiding artifacts from freezing, drying or embedding, and can in principle also be subjected to different sample environments or mechanical strains. This combination of techniques is applied to living as well as fixed and stained NIH3T3 mouse fibroblasts and the effect of the beam energy on the phase shifts is investigated. Furthermore, a 3D algebraic reconstruction scheme and a dedicated mathematical description is used to follow the motion of the trapped cells in the optical stretcher for multiple rotations.

## Abstract 2

Biological cells in suspension are manipulated using an optical stretcher and imaged using X-ray phase-contrast tomography.

## 1. Introduction

Imaging of biological cells requires probes that address the relevant lengths scales, *i.e.* nanometres to micrometres. The best-established imaging methods are without doubt fluorescence microscopy and electron microscopy (EM). Each of them offers great advantages, in particular due to recent technological advancements such as super-resolution microscopy (Hell, 2003 ▸; Shroff *et al.*, 2008 ▸) and cryo-EM (Dubochet *et al.*, 1988 ▸). X-rays offer a complementary probe with two major advantages: (i) the penetration depth of X-rays is high, thus enabling the imaging of whole, three-dimensional cells and even tissues; and (ii) using X-rays, electron density is visualized directly, thus allowing for label-free imaging. For these reasons, in recent years quite some effort has been invested into developing X-ray imaging methods that are applicable to biological samples, including single cells. Most of these methods have focused on cells adherent to substrates, which were imaged by coherent diffractive imaging (CDI) (Miao *et al.*, 2003 ▸; Nam *et al.*, 2013 ▸; Rodriguez *et al.*, 2015 ▸), scanning small-angle X-ray imaging (Weinhausen *et al.*, 2012 ▸, 2014 ▸; Bernhardt *et al.*, 2016 ▸; Hémonnot *et al.*, 2016 ▸; Nicolas *et al.*, 2017 ▸; Cassini *et al.*, 2020 ▸), ptychography (Deng *et al.*, 2017 ▸), or holography in two or three dimensions (Nicolas *et al.*, 2017 ▸; Wittmeier *et al.*, 2021 ▸, 2022 ▸). By contrast, hard X-ray imaging methods for suspension cells are rare. Notable examples are the study of frozen-hydrated *Chlamydomonas* cells by ptychographic tomography (Diaz *et al.*, 2015 ▸) and live-cell ptychography on fission yeast *Schizosaccharomyces pombe* (Strelnikova *et al.*, 2017 ▸). These examples show that the imaging of individual suspension cells in aqueous environment and in real space without ensemble averaging remains challenging. This is due to the weak difference of electron density compared with water, cell-to-cell variations, and the requirement for complex sample environments.

One approach to solving this challenge has recently been introduced by combining an optical stretcher with a dedicated X-ray setup for holotomography (Nicolas *et al.*, 2018 ▸). Optical stretchers are fiber-based two-beam traps which have initially been used to trap soft homogeneous dielectric particles (Ashkin, 1970 ▸; Constable *et al.*, 1993 ▸) but have been extended to trap and optically deform whole cells while observing them by optical microscopy (Guck *et al.*, 2000 ▸, 2001 ▸; Lincoln *et al.*, 2007*a* ▸). Nicolas *et al.* (2018 ▸) demonstrated that it is possible to use the dedicated sample environment of the optical stretcher to capture single cells, position them in the X-ray beam of a synchrotron setup, and rotate them using the flow profile in the microfluidic channel while recording holograms at multiple rotation angles.

Here, we take this idea one step further by optimizing the design of the measurement chamber of the optical stretcher. We furthermore systematically study X-ray energies, cell preparations and exposure times to obtain clearer cell images compared with previous studies. We are thus able to quantify the phase shift by the cells. The data are reconstructed both in two and three dimensions. In addition, we introduce an analysis scheme that describes the movement of the rotating cell in the optical stretcher based on Euler angles and translations. This description and an adaption of the SIRT (simultaneous iterative reconstruction technique) algorithm is used to obtain a 3D reconstruction of the electron density of grains that are used as fiduciary markers.

## 2. Materials and methods

### 2.1. X-ray phase-contrast imaging and tomography

**Figure 1:**
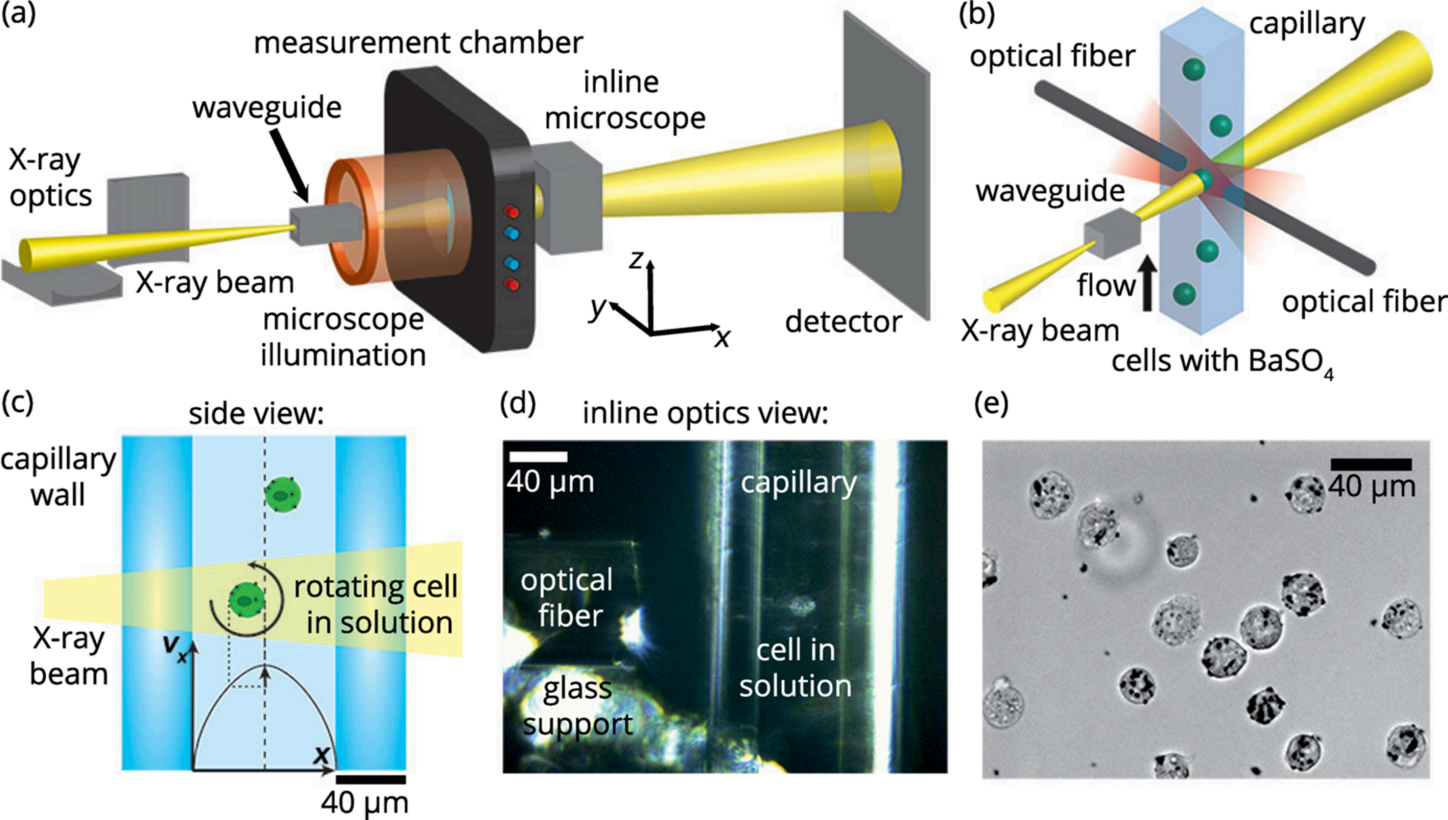
(*a*) Schematic illustration of the experimental setup at P10, PETRA III (DESY, Hamburg, Germany) including X-ray optics, a waveguide, the measurement chamber of the optical stretcher, inline microscopy for visual inspection, and a 2D pixel detector. (*b*) Main components inside the measurement chamber of the optical stretcher. Two optical fibers emit divergent IR-laser radiation that captures single cells at the trapping position inside a glass capillary with quadratic cross section. The trapped cells are probed by the X-ray beam. (*c*) Side view of the glass capillary with quadratic cross section. The cells are trapped at a slight off-center position in the capillary. Therefore, the flow velocity profile induces a rotation of the cells. (*d*) Image taken with the inline microscope showing parts of a glass support, the end of one of the optical fibers, both capillary walls, and a trapped cell in solution. (*e*) Bright field microscopy image of BaSO_4_ grains (black dots) that are assumed to be located on the surface of non-adherent NIH3T3 fibroblasts.

To combine cell manipulation using an optical stretcher and X-ray phase-contrast imaging (XPCI) and tomography (XPCT), we mount the measurement chamber of the optical stretcher in the vertical orientation in the GINIX setup (Salditt *et al.*, 2015 ▸) at P10, PETRA III (DESY, Hamburg, Germany), as sketched in Fig. 1 ▸(*a*). In this configuration the off-center trapping position of the cell is displaced towards the waveguide with respect to the center of the capillary [Figs. 1 ▸(*b*) and 1(*c*)]. X-ray holograms are recorded in cone-beam geometry at two different energies: 9.9 keV and 13.8 keV. Kirkpatrick–Baez (KB) mirrors are used to focus the X-ray beam into the entrance opening of dedicated waveguides. Waveguides provide a clean, spherical wavefront, serve as mode filter and thus decrease wavefront aberrations and increase spatial coherence (Osterhoff & Salditt, 2011 ▸; Salditt *et al.*, 2015 ▸). The waveguide used for 9.9 keV is etched into silicon using electron-beam lithography and reactive ion etching, and sealed with another layer of silicon (Eulitha, Würenlos, Switzerland) (Neubauer *et al.*, 2014 ▸). It has a cross section of approximately 110 nm × 110 nm and a channel length of 1 mm. The waveguide used for 13.8 keV is fabricated similar to the description by Krüger *et al.* (2010 ▸, 2012 ▸) but possesses a 59 nm carbon guiding layer between two 30 nm molybdenum layers in germanium and a channel length of 600 µm. Our cells are approximately twice as large (diameter ∼20 µm) as the macrophages (∼10 µm) that were studied previously at a distance of 20 mm (Nicolas *et al.*, 2018 ▸). Based on the experience with the measurement on macrophages and to take into account the larger cell diameter of the NIH3T3 cells in comparison with the macrophages and the X-ray beam, we choose a distance of 40 mm between the exit opening of the waveguide and the glass capillary. This comes at the expense of a halved magnification and a reduced photon flux on the cell, and the distance should be optimized in future experiments. The transmitted wavefronts are recorded with a Zyla detector (ZYLA5.5X-FOR, Oxford Instruments – Andor, Belfast, Northern Ireland; 2160 × 2560 pixels; pixel size: 6.5 µm × 6.5 µm) at a distance of approximately 5.1 m behind the exit opening of the waveguide. This distance is calculated by iterative determination of the Fresnel numbers for both beam energies based on an initial guess and the overlay between the positions of the minima in the expected contrast transfer function (CTF) and the power-spectral density (PSD) of the experimental holograms. Due to the cone-beam geometry, these distances yield a magnification of *M* ≃ 125×. The effective pixel size before binning is thus approximately 50 nm. During the experiment the waveguides are aligned using a photon-counting Pilatus 300K detector (Dectris, Baden, Switzerland; 487 × 619 pixels; pixel size 172 µm × 172 µm). From these alignment measurements we obtain an integrated beam intensity that varies between 1.8 × 10^8^ photons s^−1^ and 7.5 × 10^8^ photons s^−1^. We suspect that these variations in intensity are caused by instabilities of the beamline optics. The beam from the undulators is focused by KB mirrors into the opening of the waveguide. Thermal drift of both the KB mirrors and the waveguide may slightly alter the alignment and thus affect the intensity on the detector.

We use a microfluidic system to transport the cells to the trapping position in the capillary within the measurement chamber. Due to the small dimensions of the square capillary and the incompressible fluid, laminar Poisseuille flow develops. Consequently, the velocity profile is parabolic in first approximation as shown in Fig. 1 ▸(*c*). The maximum flow velocity is found in the center of the capillary and it decreases towards the walls. Thus, a cell that is not exactly centered, but positioned slightly off-center, experiences a velocity gradient and thus starts to rotate. The off-center positioning is predetermined by the position of the laser fibers with respect to the capillary in the optical stretcher. For static XPCI experiments, *i.e.* the cell is not rotating, the flow velocity is set to 0, whereas for XPCT experiments, *i.e.* the cell is rotating, we apply a pressure-driven flow. The flow direction is from the bottom to the top in the images shown in Figs. 1 ▸(*a*)–1(*d*). The inlet is connected to a vial where the cells are kept at a concentration of 60000 to 140000 cells ml^−1^ and are agitated with a magnetic stir bar at 500 r.p.m. to avoid sedimentation. The borosilicate capillary possesses a quadratic cross section with an inner diameter of 80 µm and a wall thickness of 40 µm. At the trapping position [Figs. 1 ▸(*c*) and 1(*d*)] two optical fibers emit divergent laser radiation at 1060 nm and 300 mW per fiber (beam divergence approximately 10°) in opposing directions to trap the cells in a contactless manner. The distance between the fiber ends is approximately 300 µm. The state of the trapping and the number of trapped cells are inspected with the inline microscope of the beamline setup. An image of a trapped cell is shown in Fig. 1 ▸(*d*). To trap a single cell, a slow, pressure-driven flow is applied to transport the cell through the capillary towards the trapping position. Once the cell is trapped between the optical fibers the flow is stopped and the measurement is started.

XPCI measurements are performed without flow. We typically record data sets that consist of 110 images at an empty position (with nothing in the beam), 100 images of the trapped cells in the capillary, and 5 dark images. Note that a data set may contain up to three cells, as in some cases several cells get trapped simultaneously. The exposure time is 1 s per image. Empty images of the undisturbed beam and images with cells in the capillary are recorded in an alternating manner with a batch size of 10 images. Recordings of the capillary without a cell are obtained from measurements where the cell has left the trap during the acquisition.

XPCT requires the cells to be measured from different angles. To prepare our setup for such measurements, we apply a small pressure difference of 0.1 to 0.3 mbar between the inlet and outlet. The off-center location of the trapping position of the cells and the emerging gradients in the velocity field within the capillary result in a rotation of the trapped cells. Note that, despite the flow, some cells stop rotating during acquisition. These data sets, which contain recordings without rotation, are exploited for XPCI analysis. For XPCT every data set consists of 300 images at an empty position (with nothing in the beam), 1500 images of the rotating cell, followed by another 300 images at an empty position, and 50 images to determine the dark count of the detector. The exposure time is 300 ms for each image.

### 2.2. Cell culture

We systematically study the phase contrast achieved for different cell preparations. NIH3T3 fibroblasts (DSMZ No. ACC59) are cultured at 37°C and 5% CO_2_ in a saturated water atmosphere in DMEM-high glucose (Dulbecco’s modified Eagle medium, D6429, Sigma-Aldrich, St Louis, MO, USA), supplemented with 10% FCS (fetal calf serum, S-FBSP-EU-015, Serana, Pessin, Germany), 1% penicillin-streptomycin (10000 units ml^−1^, 15140122, Gibco, Grand Island NY, USA) and 1% glutamax (35050-061, Gibco). Note that we here perform a proof-of-concept study on a cell line that is easily accessible, well described in the literature, and has low biosafety requirements. However, the method is applicable to other cell types, such as blood cells. The cells are split approximately every two days. Once a confluence of approximately 90% is reached, the medium is discarded and the cells are washed with D-PBS (Dulbecco’s phosphate-buffered saline, D8537, Sigma-Aldrich) at 37°C. The cells are detached by incubation with 0.02% EDTA/0.05% trypsin (P10-023100, PAN Biotech, Aidenbach, Germany) at 37°C for 3 min. Trypsinization is stopped by adding fresh cell culture medium. A new passage is seeded with approximately 6.7 × 10^4^ cells ml^−1^.

### 2.3. Cell labeling and staining

#### 2.3.1. BaSO_4_ grains

Since there is no direct control over the rotation angle of the cell in XPCT measurements in the optical stretcher, we attach BaSO_4_ grains to the cells which serve as fiducial markers for the rotation of the cells [see Fig. 1 ▸(*e*)]. We mix 3 µL BaSO_4_ solution (12SA002A, Guerbet, Villepinte, France) and 15 ml of cell suspension (1.9 × 10^5^ cells ml^−1^ medium). The solution is added to a Petry dish with a diameter of 10 cm (150466, Thermo Fisher Scientific, Waltham MA, USA) and incubated at 37°C, 5% CO_2_ for 2–6 h. To remove unbound grains after incubation, the medium is removed and the attached cells are washed three times with warmed D-PBS before they are detached with 1.5 ml 0.02% EDTA/0.05% trypsin in analogy to splitting. Trypsinization is stopped by adding 1.5 ml warmed and filtered (filters: 7699822, ThGeyer, Renningen, Germany) trypsine inhibitor (T6522, Sigma-Aldrich, 0.25 g L^−1^ in D-PBS) (Cossarizza *et al.*, 2017 ▸). For living cells we dilute the samples to approximately 10^5^ cells ml^−1^ to be used in the experiments.

#### 2.3.2. Lead-hematein staining

In addition to the living cells, we prepare cell samples that are fixed and stained with lead-hematein to increase the local electron density and thus the signal during X-ray experiments. The protocol is adapted from staining nuclei in whole tissue (Müller *et al.*, 2018 ▸). To exchange liquids during the staining procedure the cell solution is centrifuged (Eppendorf 5810R, Hamburg, Germany) at 100*g* for 10 min to form a pellet. The supernatant is discarded and the pellet is dissolved in 2.5 ml warmed, methanol-free formaldehyde fixing solution (28906, Thermo Fisher, 4% in D-PBS). The fixing solution with the cells is placed on a digital tube roller mixer (LLG-uniroller 6 pro, LLG Labware, Meckenheim, Germany) at 30 r.p.m. for 15 min. To remove the fixing solution and salt residues, we wash the fixed cells once with 10 ml of isotonic (9 g L^−1^) NaCl (3957.1, Carl Roth, Karlsruhe, Germany) solution and once with ultra-pure water at 270*g* for 3 min. Staining solutions are produced as described by Müller *et al.* (2018 ▸). The resulting 0.7 *M* lead(II) acetate trihydrate (316512, Sigma-Aldrich) and 0.3 *M* hematein solutions are further diluted at a ratio of 1:49 with ultra-pure water and ethanol (9065.1, Carl Roth), respectively. First, the cell pellet is dissolved in 2.5 ml diluted lead(II) acetate trihydrate solution and incubated on the digital tube roller mixer at 30 r.p.m. for 30 min at room temperature. Afterwards, the solution is washed with 10 ml ultra-pure water (centrifugation at 270*g* for 3 min) to avoid precipitation. This procedure for lead(II) acetate trihydrate is repeated for the diluted hematein solution. To blue the stain the cell pellet is suspended in 2 ml Scott’s solution (11192.01000, Morphisto, Offenbach, Germany) for 5 min at room temperature. Finally, the cells are suspended in D-PBS, inspected under a microscope (Olympus CKX51, Olympus Europa, Hamburg, Germany) and counted. The microscope is also used for quality control during the sample preparation procedure.

### 2.4. Data analysis

#### 2.4.1. X-ray phase-contrast imaging

A detailed description of the data analysis can be found in the supporting information. In brief, for the different beam energies and sample preparations, between 5 and 12 cells are evaluated and cell-free holograms are identified, which serve as background data. The holograms are processed by correction for the exposure time, area cropping to exclude the signal from the outer capillary walls, and empty-beam correction. Furthermore, a median filter is applied to reduce image noise, low frequency artifacts are removed, and binning is used to reduce the computational efforts. We here refer to single holograms that contain the signal from the capillary and the cells by *H*_*i*,cell,cap_ and to holograms of the cell-free, liquid-filled capillary as *H*_*i*, cap_ (see Table S1 in the supporting information).

Multiple consecutively recorded holograms are normalized using a median-of-medians and are averaged to represent different exposure times between *t* = 0.3 s and *t* = 450 s, which we refer to as *H*_*t*, cell, cap, *E*_. Similarly, an average, cell-free hologram *H*_cap, *E*_ is created for every beam energy *E*. Before division the cell-free holograms are scaled to the intensity of the cell-containing holograms and are aligned with the help of debris on the capillary.

The resulting cell holograms *H*_*t*, cell, *E*_ are further cropped to a rectangle containing the cell, its holographic fringes and a homogeneous background signal close to 1. The corresponding phase shifts are computed using the results from an initial contrast-transfer-function (CTF) based reconstruction as input for the relaxed averaged alternating reflection (RAAR) algorithm. Both reconstructions are constrained by a multi-area support and a maximum phase shift of ϕ_max_ = 0 rad. Finally, we estimate the noise level in the reconstructions for different exposure times.

#### 2.4.2. X-ray phase-contrast tomography

We demonstrate a procedure to obtain a tomographic reconstruction of the BaSO_4_ grains on the surface of a single cell, acquired at 9.9 keV. CTF reconstructions of the cell are obtained similarly to the XPCI analysis. We segment the BaSO_4_ grains in the reconstructions and determine their center of mass. These coordinates are manually indexed to track the motion of the grains in different projections.

To describe the rotational motion of the cells through the grain coordinates, we use the following assumptions: (i) The BaSO_4_ grains are considered to be static and located on the surface of a spherical cell. (ii) The cellular motion is described by three Euler angles, a translation along the *y* axis and constant offsets in the *x* and *z* directions in the laboratory frame. (iii) Rotation matrices and translations translate the BaSO_4_ grain coordinates from the cell to the laboratory frame. This description offers the possibility to analyze large data sets with multiple rotations at once. We formulate the optimization of the description to the experimental data as an inverse problem with regularizations, *i.e.* small changes between consecutive projections and a spherical cell shape are favored, and solve it with simulated annealing.

A 3D reconstruction of the grains is obtained by applying an adaption of SIRT to the projections that contain the segmented phase shift of the BaSO_4_ grains. Finally, the 3D phase shift is translated into a 3D electron density.

## 3. Results and discussion

### 3.1. Characterization of the X-ray compatible optical stretcher

Our X-ray compatible optical stretcher is based on a layout described in detail elsewhere (Lincoln *et al.*, 2007*a* ▸,*b* ▸). Briefly, the cells are delivered in a microfluidic capillary to the trapping location. The trapping is achieved by two counter-propagating divergent 1060 nm Gaussian laser beams of 300 mW power per fiber and guided to the trapping site with two optical single mode fibers [Figs. 1 ▸(*b*) and 1(*d*)]. In the original design, the fibers and the capillary are kept in place by supporting layers, *i.e.* an index matching gel and several layers of glass. Although this sandwich design is well suited for microscopy and has been successfully used to investigate macrophages with XPCT (Nicolas *et al.*, 2018 ▸), it absorbs parts of the X-radiation and thus decreases the measured intensities. Therefore, in our optimized design, we remove the index matching gel and all the glass layers except for the liquid-filled glass capillary from the beam path [see Fig. 1 ▸(*b*)]. Moreover, we include holes close to the capillary where the beam can propagate through without disturbance and without moving the stage-mounted measurement chamber far. The recordings of the undisturbed wavefront are necessary for the empty-beam correction during the analysis (Salditt *et al.*, 2015 ▸).

**Figure 2:**
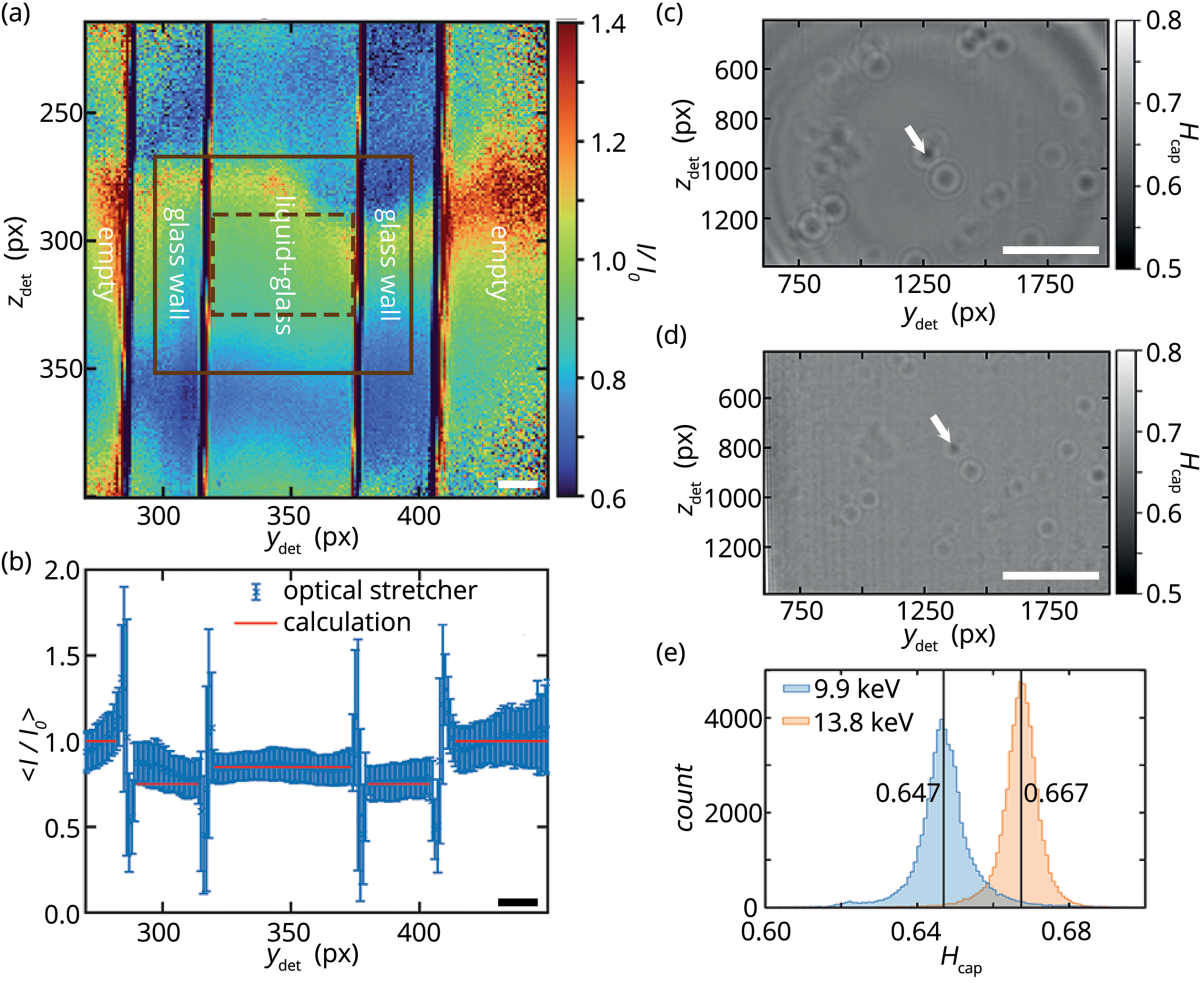
(*a*) The empty-beam-corrected intensity measured on the detector *I*/*I*_0_ for a beam energy of 13.8 keV with the capillary of the optical stretcher in the beam is determined from two 1 s exposures with the Pilatus detector during waveguide alignment. The spatial axes are given in pixels (px). Visible are the liquid-filled inner part and the walls of the quadratic glass capillary as well as empty areas around the capillary. The brown solid and dashed rectangles represent the field of view of the Zyla detector and its used area, respectively. Scale bar: 20 µm. (*b*) Empty-beam-corrected intensity profile for 13.8 keV with mean values along the individual columns in (*a*). The red lines represent calculated values based on the dimensions of the capillary. The capillary walls show slightly lower intensity than the inner part of the capillary. Scale bar: 20 µm. (*c*, *d*) Average holograms *H*_cap, *E*_ of the liquid-filled capillary without cells which are recorded with the Zyla detector. The field of view is represented by the dashed rectangle in (*a*). Scale bars: 20 µm. The dark spots stem from phase-shift and absorption that is generated by debris on the inner or outer surfaces of the capillary (see white arrows for an example). (*c*) Two data sets with in total 353.1 s exposure time are normalized and averaged to represent the average background hologram of the empty capillary for 9.9 keV. (*d*) In analogy, in total 351.6 s exposure time are normalized and averaged for a photon energy of 13.8 keV. (*e*) Distributions of the empty-beam-corrected intensities in the average background holograms for both photon energies, 9.9 keV and 13.8 keV, as shown in (*c*) and (*d*), respectively. For better visualization, outliers are not included in the plots. The vertical lines represent the median values of the distributions.

During our measurements, the only object that remains in the beam path and thus interacts with the X-ray wavefront is the liquid-filled glass capillary. Fig. 2 ▸(*a*) shows a empty-beam-corrected image of the capillary for a beam energy of 13.8 keV. We are aware that the intensities measured on the detector are not pure transmission values, but contain modulations that are visible as fringes at the edges of (sub)structures of the sample. These fringes result from the self-interference of the primary X-ray beam with diffracted waves of the sample at the detector plane. Nevertheless, we compare the measured intensities with calculated transmission values. We use the mass attenuation coefficient μ/ρ (Henke *et al.*, 1993 ▸; Salditt *et al.*, 2017 ▸). For water and borosilicate glass, mass attenuation coefficients are listed for 8 keV, 10 keV and 15 keV (Hubbell & Seltzer, 2004 ▸) and are linearly interpolated to our beam energies. With these values, as well as the mass densities of borosilicate glass (ρ_glass_ = 2.23 g cm^−3^) and water (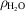 = 1.0 g cm^−3^), the path lengths in the capillary, and using Lambert–Beer’s law the transmissions through the wall*T*_cap, wall_ and the center *T*_cap, center_ of the capillary are calculated. Approximately 53% and 75% of the X-radiation is transmitted through the capillary walls for 9.9 keV and 13.8 keV, respectively. Our calculation of the transmission through the liquid-filled inner part of the capillary yields approximately 69% for 9.9 keV and 85% for 13.8 keV. As expected, X-ray photons with higher beam energy interact less with the matter and thus a higher transmission is achieved.

The calculated transmission values and line averages from the experimentally obtained empty-beam-corrected intensity [Fig. 2 ▸(*a*)] agree well with each other, see Fig. 2 ▸(*b*). The intensity is approximately 1.0 outside of the capillary, where the beam propagates only through air without disturbance. Lower intensity is observed for passing through the 160 µm glass walls than for passing through the central liquid-filled part of the capillary. Due to measurement artifacts such as beam intensity fluctuations and thermal drift, we observe areas in Fig. 2 ▸(*a*) where *I*/*I*_0_ > 1.0 which should not be observed for a steady beam and stable optics.

As described in Section 2.4 and in more detail in the supporting information, average holograms of the liquid-filled part of the capillary are calculated for both beam energies to serve as background. These holograms are shown in Figs. 2 ▸(*c*) and 2(*d*). Clearly visible are dark spots in both images (see white arrows for an example), which originate from debris on the outer surface of the capillary. Due to the change in wavelength and thus in Fresnel number, these spots are larger for 9.9 keV compared with 13.8 keV. We can make use of these signals for alignment of different images. Fortunately, the debris does not disturb the holograms of the cells because it is present in the average background holograms and in the holograms of the cells and background. Therefore, the signal of the debris cancels out during background division.

The empty-beam-corrected intensity values of the average background holograms for both beam energies are presented as histograms in Fig. 2 ▸(*e*), along with their median values. Interestingly, the calculated value (69%) for 9.9 keV agrees well with the experimentally obtained transmission (65%), whereas this is not the case for 13.8 keV (85% *versus* 67%). The reason for this discrepancy is likely found in intensity fluctuations during the measurements. Indeed, we observe variations of the image intensity within and between different data sets for both the unprocessed and empty-beam-corrected acquisitions. Since acquisitions of the undisturbed wavefront are recorded only before or after the acquisitions of the cell with a total exposure time of 450 s, thermal drift of the waveguide cannot be ruled out. For this reason, the empty-beam-corrected holograms are normalized with their median-of-medians before averaging (see supporting information).

### 3.2. Influence of the exposure time

**Figure 3:**
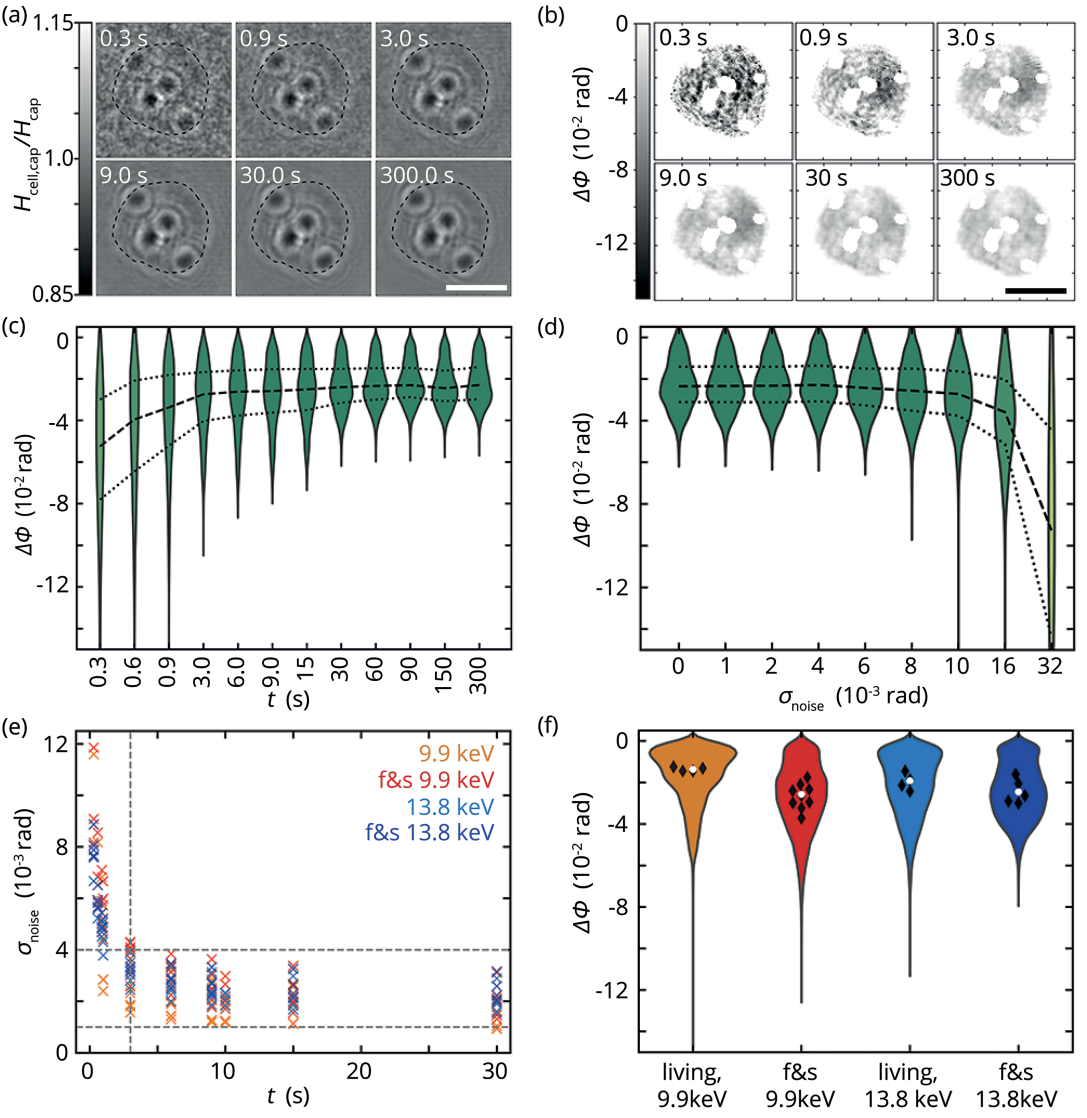
(*a*) Background-corrected holograms from a single data set of a fixed and stained cell, measured at 9.9 keV and with different accumulated exposure times between 0.3 s and 300 s, demonstrate the noise level of the recordings. The dashed lines represent the chosen reconstruction support. Scale bar: 10 µm. (*b*) Reconstructions using the RAAR algorithm based on holograms with different exposure times for the same data set as in (*a*). BaSO_4_ grains are excluded after the reconstruction. Scale bar: 10 µm. (*c*) Violin plots of the reconstructed phase shift from (*b*) for different exposure times. To guide the eye, lines are drawn for the 25% (dotted), 50% (dashed) and 75% (dotted) quantiles. (*d*) Phase shift distribution from reconstructions of noisy holograms of the sample from (*a*–*c*). Gaussian noise with amplitudes σ_noise_ is added to the hologram with an exposure time of 30 s and reconstructed. Quantiles for 25% (dotted), 50% (dashed) and 75% (dotted) guide the eye. (*e*) Standard deviation of the noise in the holograms. Data from fixed and stained (f&s, red and dark blue) and from living cells (orange and light blue) for both photon energies are shown. (*f*) Violin plots of the reconstructed phase shifts of the cytoplasm (without BaSO_4_ and debris) for an exposure time of 30 s. Within the violins the medians of the individual data sets (black diamonds) and the overall distribution (white circle) are shown.

Like any soft and biological matter, cells are sensitive to radiation damage. Imaging cells with X-rays therefore poses a great challenge: the exposure time needs to be long enough to provide sufficient contrast but should not exceed this value so as not to interfere with cell integrity. We systematically investigate the exposure time necessary for obtaining meaningful data from X-ray holography within the optical stretcher. Fig. 3 ▸(*a*) shows holograms of a non-rotating, fixed and stained cell measured at 9.9 keV for different accumulated exposure times. For long exposure times clear holographic fringes are visible that correspond to the outline of the cell. However, with decreasing exposure time the noise level increases, concealing the cell outline below approximately 3 s. Outside the cell, the holograms possess pixel values of approximately 1, indicating that the background is properly removed, and thus only the signal of the cell is contained in the holograms. Although the cell outline vanishes for low exposure times the holographic fringes of the BaSO_4_ grains remain visible at the shortest exposure time of 0.3 s. Therefore, they can also serve as fiducial markers in holograms with high noise levels.

Since the cell outline is visible already in the holograms, the support for the RAAR reconstruction can easily be chosen for fixed and stained samples that are measured at 9.9 keV. Similar to the holograms, the RAAR reconstructions show a dependency of the phase shift of the cell cytoplasm on the exposure time [see Figs. 3 ▸(*b*) and 3(*c*)]. In comparison with longer exposure times, short exposure times lead to larger (negative) phase shifts and a wider spread of the phase shift distribution of the cytoplasm. A constant median and distribution of the phase shift is observed for exposure times of more than ∼3 s, which is a typical observation for all the samples of this study. Effects from the choice of parameters for the RAAR algorithm are unlikely to influence this result since the algorithm does not depend on regularization parameters but only on the number of iterations. A value of 500 iterations has been successfully used before (Nicolas*et al.*, 2018 ▸).

The reconstructed cytoplasm features speckle-like patterns [see Fig. 3 ▸(*b*)] which we assign to noisy patterns in the holograms for small exposure times. We therefore hypothesize that the noise in the holograms influences the phase shift in the reconstructions. To test this assumption, we investigate the influence of additive Gaussian noise with different amplitudes σ_noise_ on holograms with exposure times of 30 s [see Fig. 3 ▸(*d*)]. We employ Gaussian noise here to simplify the approach. As expected, the obtained phase shift increases and spreads over a larger range, as similarly observed for the reconstructions with small exposure times. The effect becomes strongly visible for σ_noise_ > 0.01, which is larger than the noise amplitudes in the experimental data [see Fig. 3 ▸(*e*)]. Nevertheless, additive Gaussian noise is still able to qualitatively reproduce the observed phase shifts and thus demonstrate the influence of the noise level on the phase shifts in the reconstructions. A high noise level in a hologram due to a small exposure time leads to large phase shifts in the associated reconstruction.

The experimentally obtained amplitudes of the noise in the holograms are shown in Fig. 3 ▸(*e*) for all the samples and for the first 30 s of exposure time. The noise decreases with increasing exposure time and reaches a plateau between 10^−3^ rad and 4 × 10^−3^ rad (gray horizontal dashed lines). However, the procedure to estimate the noise amplitudes in the holograms by using a 2D quadratic fit might not completely capture the shape of the holographic fringes and the complex noise distribution in the holograms.

The cells are exposed to both X-rays and IR radiation. The maximum dose imposed by the X-rays can be estimated according to Shen *et al.* (2004 ▸) by assuming a mass density of ρ = 1.35 g cm^−3^, and cellular contents that are modeled as H_50_C_30_N_9_O_10_S_1_ (Howells *et al.*, 2009 ▸). The assumption of surface dose slightly overestimates the average dose in the object and hence represents an upper bound. Attenuation lengths of 1.4 mm (9.9 keV) and 3.75 mm (13.8 keV) (Henke *et al.*, 1993 ▸), a maximum flux on the sample of 5.7 × 10^4^ photons s^−1^ µm^−2^ that is obtained from a Pilatus acquisition of an empty beam on the detector, and an exposure time of 1500 × 0.3 s yield maximum doses of approximately 22 kGy (9.9 keV) and 12 kGy (13.8 keV). Indeed, the flux is further attenuated by 40 µm glass and approximately 25 µm water before it reaches the cell which can be calculated in analogy to Section 3.1. This results in doses on the cell in the capillary of the optical stretcher of 18 kGy (9.9 keV) and 10 kGy (13.8 keV). This dose is orders of magnitude below doses that have been reported for X-ray scattering experiments (Henderson, 1990 ▸; Howells *et al.*, 2009 ▸) and we thus consider the method rather ‘gentle’ for the cells.

IR radiation affects the cells by heating the water inside and outside of the cell. By contrast, proteins barely absorb at the trapping wavelength of 1060 nm (Lincoln *et al.*, 2007*a* ▸; Wetzel *et al.*, 2011 ▸). Typical slopes for the temperature increase with respect to the total irradiated power are 10–13 K W^−1^ for the ‘classical’ optical stretcher design. The temperature increase is effectively realized within milliseconds, followed by a very flat plateau-like increase over time (Ebert *et al.*, 2007 ▸; Wetzel *et al.*, 2011 ▸; Kießling *et al.*, 2013 ▸). In our experiment 2 × 0.3 W are used yielding a temperature increase of Δ*T* ≃ 8 K. Therefore, the IR radiation of the trap heats our cells from ambient conditions to approximately 28°C, which is within a suitable temperature range for living cells. We expect similar heating with or without index matching gel, which is usually used in optical stretchers, because it effectively does not absorb at 1060 nm (G608N3, Thorlabs, Newton, NJ, USA).

### 3.3. Influence of cell staining

Apart from the exposure time, the sample preparation may influence the quality of the holograms. The ultimate goal is to image living cells; however, we benchmark those data against fixed and stained samples for improved contrast. We therefore compare the phase shifts for the different sample categories for an exposure time of 30 s, which is available in most of the considered data sets. Fig. 3 ▸(*f*) shows violin plots of the phase shift distribution within the reconstruction support without the signal of the BaSO_4_ markers, *i.e.* the phase shift obtained for the cytoplasm. The negative phase shift is slightly larger for the fixed and stained samples than for the living cells.

**Figure 4:**
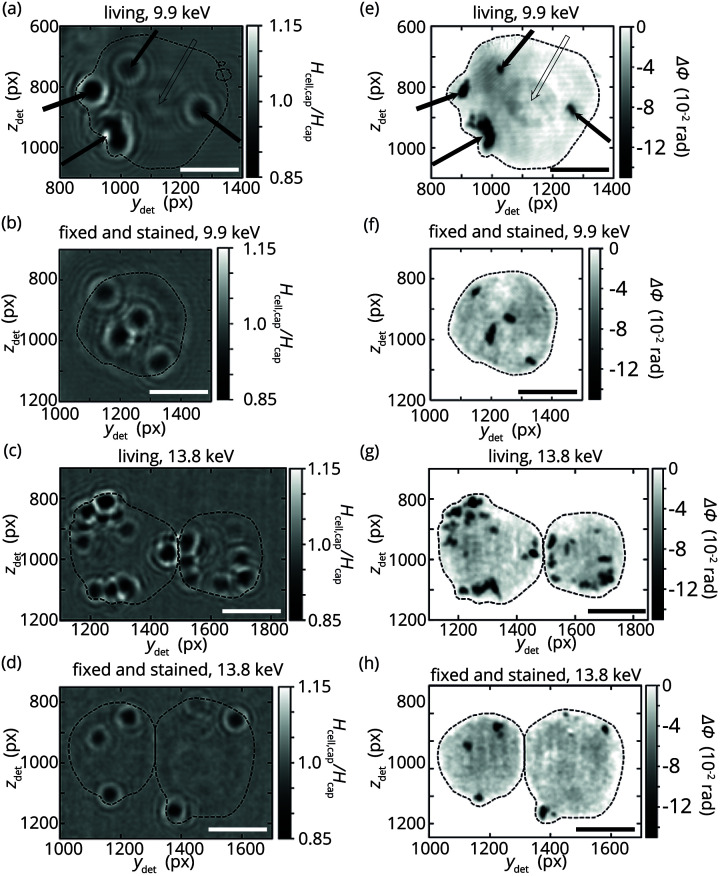
(*a*–*d*) Background-corrected holograms of representative living, as well as fixed and stained cells for 9.9 keV (*a*, *b*) and 13.8 keV (*c*, *d*). The dashed lines represent the outline of the reconstruction support (see Fig. S2 in the supporting information for a version without the dashed lines). The exposure time for the holograms is 30 s. Black dots in the holograms and reconstructions represent BaSO_4_ grains (see black filled arrows for examples) that are attached to cells. Scale bars: 10 µm. (*e*–*h*) Reconstructed phase shift caused by the holograms in (*a*–*d*). A CTF reconstruction is used as initial phase guess which is refined using the RAAR algorithm with 500 iterations. To improve visibility of the reconstructed nucleus and cytoplasm, the phase shift from BaSO_4_ grains goes beyond the chosen color scale of these plots. (*e*) The cell nucleus is visible in some reconstructions of living cells that are recorded at 9.9 keV (see black open arrow). The spatial axes are given in pixels (px).

The effects of different cell preparations and beam energies can also be observed in the holograms and the reconstructions of the cells (see Fig. 4 ▸). While the cell outline is visible in the holograms for fixed and stained samples, it can only be estimated for the living samples (see Fig. S2 in the supporting information for a version without the drawn reconstruction support). Similarly, the associated reconstructions show sharper cell boundaries for fixed and stained samples than for living samples. Since the reconstruction support is chosen based on the cell outline, this choice is more reliable for fixed and stained samples. We attribute this result to the lead component of the lead-hematein staining, which increases the local electron density and thus causes a stronger signal in the holograms. The lead-hematein stain has successfully been used to stain nuclei in tissue blocks and associates with the negative charges, *e.g.* in the DNA backbone (Müller *et al.*, 2018 ▸). Interestingly, however, in our single cell experiments of stained samples, we cannot distinguish the nucleus from the cell cytoplasm. This may be caused by insufficient quantities of staining agent in the nucleus, due to too short staining times. Alternatively, insufficient protonation of negatively charged molecules in the cytoplasm or overly long staining times might have caused an overstaining of the cytoplasm and thus reduced the contrast between cytoplasm and nucleus. Despite this need for optimization of the staining procedure, we are able to show that the X-ray stain increases the phase shift of the cells in the optical stretcher compared with living cells, especially for a photon energy of 9.9 keV [see Fig. 3 ▸(*f*)].

In all holograms and reconstructions we clearly see the BaSO_4_ grains. By contrast to the macrophages studied by Nicolas *et al.* (2018 ▸), we assume that the fibroblasts we study do not engulf these grains, but they are attached to the cell surface and act as fiducial markers. Note that a spherical shape of the cell bodies would be expected to be visible in the reconstructions. This shape requires low spatial frequencies, which only weakly contribute to the phase contrast in measured holograms. Low frequencies can more easily be deduced from absorption contrast, which is very low in these experiments and thus may be concealed by the strong noise level in the acquired images. For this reason, reconstruction of the low spatial frequencies is difficult and thus the spherical shape of the cells is not as visible as expected.

### 3.4. Influence of the photon energy

The X-ray photon energy for holography on cells needs to be chosen with care. At higher energy the X-rays more readily penetrate the glass capillary walls and the liquid inside the capillary, whereas at lower energy they are absorbed more by the cells in the beam [see Figs. 2 ▸(*c*)–2(*e*)]. Moreover, the interactions of the X-ray photons with the stain and the grains differ with energy. We therefore compare data taken at 9.9 keV and 13.8 keV. Interestingly, the 9.9 keV data for living cells, but not the other conditions, reveal the cell nucleus [see open arrow in Figs. 4 ▸(*a*)–4(*e*)]. We observe this clearly separated nucleus for about half of the data sets for living cells at 9.9 keV. When investigating fixed and stained cells we detect a sharp cell outline [see Figs. 4 ▸(*b*) and 4(*f*)] and we observe the largest overall phase shift in the cytoplasm for 9.9 keV [see Fig. 3 ▸(*f*)]. The latter can be explained with the β/δ ratio for lead, which is 8.4 × 10^−2^ for 9.9 keV and thus smaller than 1.2 × 10^−1^ for 13.8 keV (Henke *et al.*, 1993 ▸) while the mass attenuation is similar for both energies (136.3 cm^2^ g^−1^ for 9.9 keV, 142 cm^2^ g^−1^ for 13.8 keV) (Hubbell & Seltzer, 2004 ▸). This leads to a stronger phase shift for 9.9 keV measurements. We thus conclude that a photon energy of 9.9 keV is a good choice for X-ray holography measurements with the X-ray compatible optical stretcher.

To evaluate the changes we performed on the measurement chamber, *i.e.* the removal of layers of glass and index matching gel, we compare our results of living NIH3T3 cells, measured at 13.8 keV, with the available data that are reported for fixed macrophages that are also measured at 13.8 keV using the original version of the optical stretcher (Nicolas *et al.*, 2018 ▸). It is not expected that the chemical fixation increases the contrast because it does not insert electron-rich elements into the cell. Therefore we compare our living cells with the previously reported, unstained fixed cells. Both measurements have in common that the contour of the cells cannot be identified in the single projections. A quantitative comparison is not possible due to missing noise levels but the following scaling argument applies. In the work by Nicolas *et al.* (2018 ▸) a 10–40× higher intensity was used, resulting in a 10–40× higher photon flux. In that work, the waveguide is located at half the distance, increasing the flux on the sample by a factor of 4 (2 × 2, as it scales with the area); thus in total we need to scale the exposure time of 0.1 s by 40–160 (resulting in 4–16 s) in order to compare with our minimum exposure times of 3 s. We can thus conclude that the adaptations in the design of the optical stretcher improve the quality of the obtained data.

### 3.5. X-ray holo-tomography

The flow in the capillary of the optical stretcher offers the unique possibility to rotate the trapped cells by merely positioning them slightly off-center within the Poiseuille flow (Nicolas *et al.*, 2018 ▸). The continuous flow has the additional advantage of cooling of the cell, thus diminishing cell damage. To stably trap a cell in constant flow, due to the additional Stokes drag force, we increase the trapping laser power to 0.3 W per fiber, which is below values where deformations of eukaryotic cells are observed (Guck *et al.*, 2001 ▸). We operate our optical stretcher at a slightly lower cell concentration of 10^5^ cells ml^−1^ instead of the typically used 2 × 10^5^ to 5 × 10^5^ cells ml^−1^ (Lincoln *et al.*, 2007*a* ▸,*b* ▸) to avoid too many additional cells being flushed into the trapping region.

**Figure 5:**
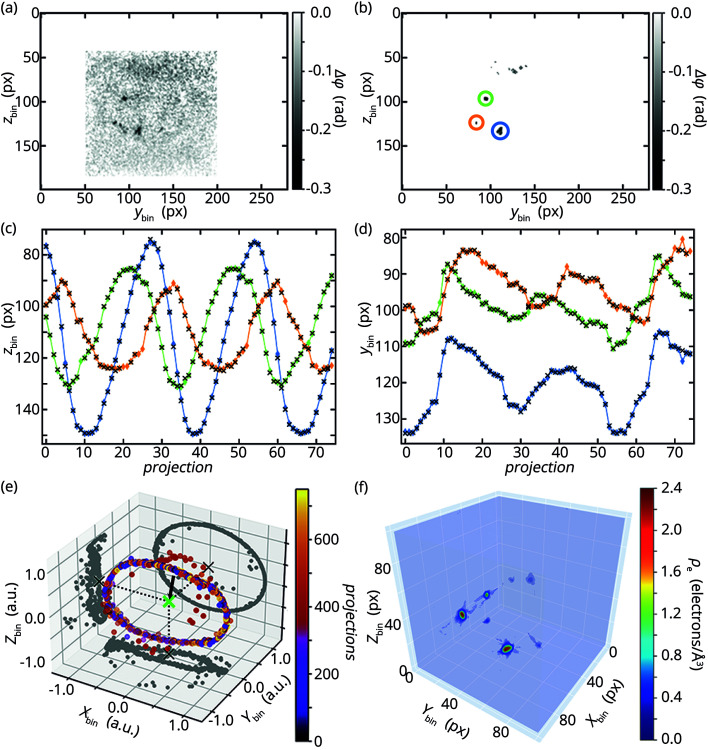
(*a*) Noisy CTF reconstruction from a single projection (hologram, binning: 5 × 5). The faint boundary of the cell and strong signals of BaSO_4_ grains are visible. The spatial axes are given in pixels (px). (*b*) The grains from (*a*) are segmented with a median filter and a threshold. A selection of BaSO_4_ grains is manually indexed in every projection, such that the movement of these grains can be tracked over multiple projections. Grains with different indices are encircled in different colors. (*c*, *d*) Tracked *y* and *z* coordinates (center of mass, black crosses) of the segmented and indexed grains in 75 out of 750 projections. In addition, the calculated *y* and *z* coordinates (**r**_*i*,*n*,calc_, colored diamonds) of the indexed grains from the solution of the inverse problem formulated to determine the Euler angles and translations are plotted for different projections. To guide the eye, the calculated coordinates are colored according to their index and are connected by a line. (*e*) Direction of the X-ray beam for 750 projections in the frame of reference of the cell. Since the cell rotates in the trap, the beam direction, *i.e.* the arrow from every dot to the center of mass of the cell (green cross), changes from projection to projection. Additionally, projections to the different planes are shown. (*f*) SIRT-based 3D reconstructions of the electron density of BaSO_4_ grains.

The drawback of rotation by flow, however, is that the rotation of the cell cannot directly be controlled and needs to be determined from the projections. For this purpose, we use the BaSO_4_ grains as fiducial markers which are clearly visible against the background noise [see Fig. 5 ▸(*a*)] and thus can be easily segmented and indexed [see Fig. 5 ▸(*b*)]. The projected movement of the indexed markers, *i.e.* the tracks of the center of mass, perpendicular to the beam in the vertical (*z*) and horizontal (*y*) direction are shown in Figs. 5 ▸(*c*) and 5(*d*). In the *z* direction the coordinates of the markers follow an oscillatory movement which is not completely sinusoidal in contrast to what is reported for the macrophages (Nicolas *et al.*, 2018 ▸). Based on observations from test experiments some of the NIH3T3 cells in solution deviate from the spherical shape. Therefore, the velocity gradient, and thus the torque acting on the trapped cell, changes as a function of the rotation angle. As a consequence, the angular velocity changes.

Our cells and the macrophages have in common that they move in parallel to the laser beam axis, *i.e.* the *y* axis, in the optical stretcher which is visible in Fig. 5 ▸(*d*). In this example, the average movement range of the three indexed markers in the *y* direction spans *t*_*y*_ = 58 ± 7 binned pixels in 750 projections [see Fig. 5 ▸(*d*)], which corresponds to 15 ± 3 µm in the trap assuming an error of 10% for the magnification. Nevertheless, the good agreement of the calculated coordinates (colored diamonds, solid lines) from the optimization and the experimentally obtained coordinates (black crosses) in Figs. 5 ▸(*c*) and 5(*d*) demonstrates that we can describe the motion of the cell in the optical stretcher. The obtained Euler angles and translations are shown in Fig. S3 in the supporting information.

Fig. 5 ▸(*e*) and Figs. S4(*a*)–S4(*c*) in the supporting information offer another way to visualize the rotation by showing the direction of origin of the X-ray beam in the frame of reference of the cell which is calculated by [*M*(α_*n*_)*M*(β_*n*_)*M*(γ_*n*_)]^−1^(1, 0, 0)^*t*^. Clearly, a circle is visible in the *XZ* plane indicating a major rotation axis *Y*. The projections into the other two planes deviate from the straight line that would be expected if only one axis of rotation was present. Therefore, rotations around multiple axes contribute to the overall rotation which can be explained by two properties of the cell in the optical stretcher. First, due to the flow velocity field in the quadratic capillary the cell is not just influenced by a velocity gradient along the *x* direction, which causes the previously described major rotational movement, but also a velocity gradient along the *y* direction, which vanishes only in the center of the *y* axis in the capillary. Therefore, the observed translation along the *y* direction results in an additional torque component. Second, cells in an optical stretcher align their axis of largest optical anisotropy in parallel to the optical axis (Kreysing *et al.*, 2008 ▸), *i.e.* the *y* axis in our experiment. Optical anisotropy of biological objects like cells is rooted in their heterogeneous composition (*e.g.* the positioning of the nucleus and other organelles), which results in a heterogeneous refractive index and therefore different speeds of light in different directions. Due to the complexity of the cell, we assume that multiple axes with a large optical anisotropy exist and small disturbance of the flow field during the experiment could cause the cell to change its orientation with respect to the laser axis.

Using the rotation angles and translations from our transformation function between the reference frame of the cell and the laboratory, it would in principle be possible to reconstruct the 3D shape of the entire cell. However, the noise levels in the reconstruction of the single projections are too high in our experiment [see Fig. 5 ▸(*a*)]. Therefore, we focus on reconstructing the BaSO_4_ grains [see Fig. 5 ▸(*f*)] for our example data set, which exhibit a stronger signal. Shown is the result after 200 iterations with the SIRT algorithm where every voxel of the 3D reconstruction is divided into eight sub-volumes. To evaluate the 3D reconstruction, we calculate the sum of squared differences between the forward projections of the 3D reconstruction for a given number of iterations and the segmented BaSO_4_ grains for every projection. The average over all sums reaches a value of 0.99 rad^2^ with an empirical standard deviation of 0.61 rad^2^ and is almost constant, *i.e.* changes by less than 10^−4^ rad^2^ between iterations, for 200 iterations. Note that forward projections and experimental projections are both showing phase shifts. Higher numbers of sub-volumes increase the computation time but do not improve this measure [see Fig. S4(*d*) in the supporting information]. Despite some small artifacts, which may result from noise or under-sampled angles, the grains are clearly visible in the 3D reconstruction of the electron density. Using the parameters of the orthorhombic unit cell of crystalline BaSO_4_, *a* = 8.884 Å, *b* = 5.456 Å, *c* = 7.157 Å, the number of molecules per unit cell *Z* = 4 (James & Wood, 1925 ▸; Hill, 1977 ▸) and the number of electrons in a single BaSO_4_ molecule *n*_e_ = 104, the expected electron density of BaSO_4_ amounts to ρ_e_ = *Zn*_e_/(*abc*) ≃ 1.2 electrons Å^−3^. The experimentally obtained values in Fig. 5 ▸(*f*) yield larger electron densities by about a factor of approximately two, because the segmented phase shift of the grains in the used projections contain signal from noise, the cell, and BaSO_4_. By segmenting the grains in the projections before reconstruction, these signals are only assigned to the BaSO_4_ grains during tomographic reconstruction. As a consequence, the electron density is overestimated. Nevertheless, our reconstructed electron densities yield the same order of magnitude compared with the theoretically determined electron density. For comparison, an estimate of the electron density for the cell using an average biological materials H_50_C_30_N_9_O_10_S_1_ (Howells *et al.*, 2009 ▸) with ρ = 1350 kg m^−3^, molar mass *M* = 717.7 g mol^−1^ and number of electrons *n*_e_ = 389 yields an electron density of ρ_e_ = *N*_A_*n*_e_ρ/*M* ≃ 0.44 electrons Å^−3^. Here, *N*_A_ is Avogadro’s constant and additional contributions from the lead-hematein staining are not considered.

## 4. Conclusions

In summary, we improve the design of the measurement chamber of the optical stretcher by removing layers of glass and index matching gel, thus rendering it more compatible with X-ray measurements and diminishing the required exposure times. We apply this setup to measure non-rotating and rotating NIH3T3 fibroblast with XPCI and XPCT, respectively. We systematically investigate different experimental parameters, *i.e.*X-ray energies, exposure times and sample preparations. We find that an energy of 9.9 keV leads to a stronger phase shift if the cells are lead-hematin-stained and thus reveals sharper cell boundaries. Interestingly, in living cells it is possible to detect the nucleus. Additionally, we demonstrate that the Poisseuille flow in a capillary may be used to rotate a cell while performing tomography experiments on it. We use BaSO_4_ grains as fiducial markers for X-ray tomography on NIH3T3 fibroblasts. We show for a data set of a fixed and stained cell, measured at 9.9 keV, how the markers can be tracked for a large number of projections and the rotational movement of the cell can be described. The resulting Euler angles and translations from the mathematical description can in principle be used to obtain a 3D reconstruction of the cell in the future after optimization of the staining procedure. For this purpose, also less poisonous X-ray cytoplasm stains, *e.g.* eosin-Y or erythrosin-B (Busse *et al.*, 2021 ▸), might be applied.

We thus demonstrate the utility of the newly developed X-ray compatible optical stretcher for capturing, rotating and simultaneously imaging single cells. In future experiments, the additional functionalities of optical stretchers can be combined with our method, *i.e.* applying rheological strain on the soft cells and adjusting the chemical sample environment, *e.g.* drugs, chemicals or other reagents, in the microfluidic capillary.

Future improvements in waveguide optics and pre-focusing by the KB mirror may further increase the signal-to-noise ratio, and holographic recordings with pixel detector technology may also help to push the contrast limits for low contrast samples, such as hydrated cells. Above all, we expect that the proposed upgrade to PETRA IV alone without further improvements in optics or detection would give an intensity gain between one and two orders of magnitude, reducing the exposure time and hence also the blurring of holographic fringes.

## 5. Related literature

The following references, not cited in the main body of the paper, have been cited in the supporting information: Cloetens *et al.* (1999 ▸); Gordon (1974 ▸); Huhn *et al.* (2022 ▸); Justusson (1981 ▸); Kak & Slaney (2001 ▸); Lohse *et al.* (2020 ▸); Luke (2005 ▸); Natterer (2001 ▸); Osterhoff (2017 ▸); The HDF Group (1997 ▸); Van Rossum & Drake (2009 ▸); Xiang *et al.* (1997 ▸).

## Supplementary Material

supporting information pdf. DOI: 10.1107/S1600577524003618/vl5023sup1.pdf
