# Supplementary material for: X-ray phase-contrast tomography of cells manipulated with an optical stretcher
Source: J Synchrotron Radiat. 2024 Jun 11;31(Pt 4):923–35. doi: 10.1107/S1600577524003618 (PMC11226146; doi:10.1107/S1600577524003618)
Supplement: Supplementary file 1 [file s-31-00923-sup1.pdf]

Supporting Information: X-Ray Phase-Contrast Tomography of Cells Manipulated with an Optical Stretcher

Jan-Philipp Burchert,[a,b] Jasper Frohn,[a] Ulrike Rölleke,[a] Hendrik Bruns,[a] Boram Yu,[a] Sophie-Charlotte Gleber,[a] Roland Stange,[c] Madleen Busse,[d,e] Markus Osterhoff,[a] Tim Salditt[a,b] and Sarah Köster [a,b]*

[a]Institute for X-Ray Physics, University of Göttingen, Friedrich-Hund-Platz 1, 37077 Göttingen, Germany, [b]Cluster of Excellence "Multiscale Bioimaging: from Molecular Machines to Networks of Excitable Cells" (MBExC), University of Göttingen, Germany, [c]RS Zelltechnik, 94508 Schöllnach, Germany, [d]Biomedical Physics, School of Science, Technical University Munich, Boltzmannstraße 11, 85748 Garching, Germany, and [e]Munich Institute of Biomedical Engineering, Technical University Munich, Boltzmannstraße 11, 85748 Garching, Germany. .

E-mail: sarah.koester@uni-goettingen.de

1. Extended Description of the Data Analysis

Table 1. *Abbreviations use to indicate the different holograms.*

abbreviation	description
$H_{i,\text{cell,cap}}$	i^{th} hologram in a data set of a capillary with cell after empty-beam correction
$H_{i,\text{cap}}$	i^{th} hologram in a data set of a cell-free capillary after empty-beam correction
$H_{t,\text{cell,cap},E}$	average hologram from as many consecutively recorded $H_{i,\text{cell,cap}}$ of a data set to represent a total exposure time t
$H_{\text{cap},E}$	average hologram from all $H_{i,\text{cap}}$ that are recorded at beam energy E
$H_{t,\text{cell},E}$	hologram that only contains the signal of the cell, obtained by division of $H_{t,\text{cell,cap},E}$ by $H_{\text{cap},E}$

X-Ray Phase-Contrast Imaging We use the software *dada* (Osterhoff, 2017) to identify data sets with long sequences of non-rotating cells from XPCI and XPCT measurements. Data sets which show strong artifacts, *e.g.*, large $BaSO_4$ grains, are excluded from analysis. Moreover, we select the data set with the longest acquisition time for every single cell. If multiple data sets satisfy this condition, the one that is recorded first is selected. In total, 5 data sets with 5 living cells (9.9 keV), 11 data sets with 15 fixed and stained cells (9.9 keV), 5 data sets with 9 living cells (13.8 keV), 5 data sets with 12 fixed and stained cells (13.8 keV) and 5 data sets with sequences of images of the capillary without a cell contribute to the analysis. The latter serve as background data.

We process the data with self-written Python (3.7-3.10, (Van Rossum & Drake, 2009)) and Matlab (Matlab 9.3, 2017, The MathWorks, Natick, MA, USA) scripts and the Matlab HoloTomoToolbox (Lohse *et al.*, 2020) and store intermediate results in HDF5 files (TheHDFGroup, 1997). Similar to (Nicolas *et al.*, 2018), the acquisitions are corrected for their exposure time, cropped to the region between the capillary walls, and the beam profile is removed (empty-beam correction) to obtain holograms of the signal from the cells and the liquid-filled capillary $H_{i,\text{cell,cap}}$. Since XPCT and XPCI data contain different sequences of empty beam acquisitions and acquisitions of the

cells with background, the holograms are calculated with the dark noise of the detector I_{dark}, the average empty beam(s) I_{empty} (XPCI data sets) and $I_{\text{empty, pre}}$, $I_{\text{empty, post}}$ (XPCT data sets), and the number of projections i as follows:

$$\text{XPCT:} \qquad H_{i,\text{cell,cap}} = \frac{I_i - I_{\text{dark}}}{(1-i)\cdot I_{\text{empty, pre}} + i \cdot I_{\text{empty, post}} - I_{\text{dark}}}, \tag{1}$$

$$\text{XPCI:} \qquad H_{i,\text{cell,cap}} = \frac{I_i - I_{\text{dark}}}{I_{\text{empty}} - I_{\text{dark}}}. \tag{2}$$

We obtain holograms of the cell-free capillary $H_{i,\text{cap}}$ from data sets where the cell is lost during acquisition. The empty-beam corrected holograms I/I_0 are obtained by dividing the acquisition of the cell-free capillary I by the acquisition of the empty beam I_0.

The resulting holograms are smoothed with a 9×9 median filter, which reduces noise but does not blur edges in the hologram (Justusson, 1981). Additionally, low spatial frequencies are removed by subtracting a blurred version of the same image (Gaussian filter, $\sigma = 50$ pixels) and adding the mean pixel intensity (Lohse *et al.*, 2020). Finally, the holograms are binned (5×5) to reduce the computational efforts.

The holograms $H_{i,\text{cell,cap}}$ contain signal from the cell and the liquid-filled capillary. To obtain only the signal of the cells, we first determine average background holograms, *i.e.*, holograms of the liquid-filled capillary without cells, $H_{\text{cap},E}$, for both beam energies. The debris on the surface of the capillary is used to align the contributing holograms $H_{i,\text{cap}}$ for a beam energy E. Before averaging, a common pixel grid for all individual holograms is defined. Subsequently, all holograms are recalculated by bilinear interpolation to this common grid. Additionally, we normalize the holograms to the intensity of their median-of-medians, *i.e.*, a median pixel value is calculated from the median pixel values of every contributing hologram. The median-of-medians is chosen as a reference level to account for the variation in the primary beam intensity between different holograms. In contrast to averages, medians are less strongly affected by out-

liers in a data set. Therefore, the result of this normalization procedure is less strongly affected by the number of grains and debris in the holograms or primary beam intensity variations. The holograms that contain cell and background signal are similarly normalized with the median-of-medians. Different numbers of consecutively recorded, cell-containing holograms with a fixed exposure time are averaged to yield the holograms $H_{t,\mathrm{cell,cap},E}$ which represent different total exposure times t between 0.3 s and 450 s. To finally remove the background signal from $H_{t,\mathrm{cell,cap},E}$, the average background hologram with the same beam energy E is aligned, interpolated in a bilinear manner and scaled by $s = \frac{median(H_{t,\mathrm{cell,cap},E})}{median(H_{\mathrm{cap},E})}$ before division: $H_{t,\mathrm{cell},E} = \frac{H_{t,\mathrm{cell,cap},E}}{s \cdot H_{\mathrm{cap},E}}$.

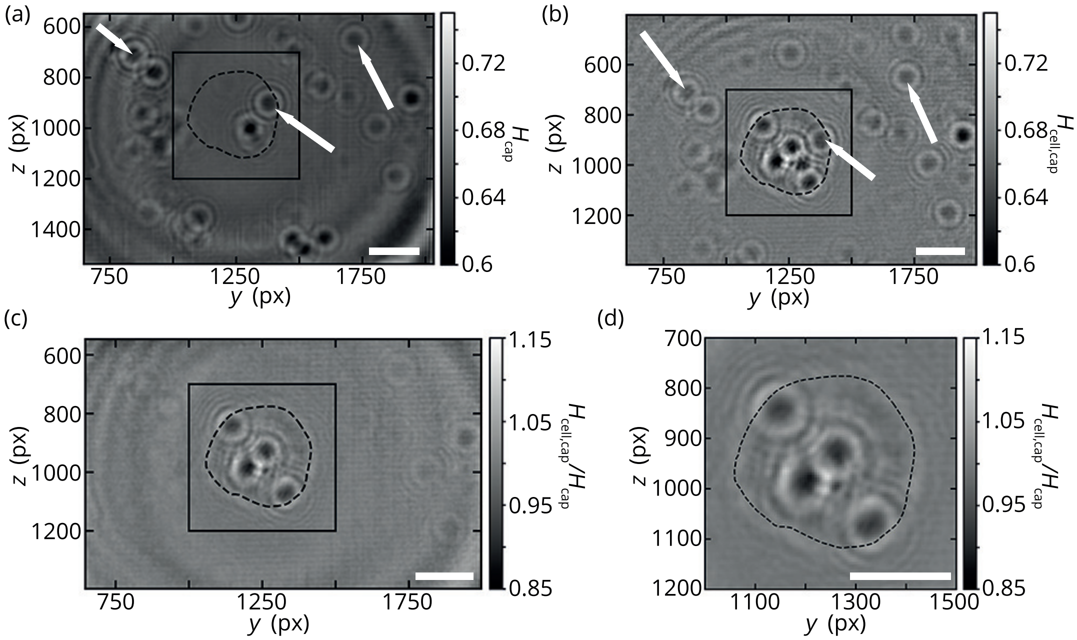

Fig. S1. Step-by-step explanation of the analysis procedure of the holograms shown for a lead-hematein-stained cell that is measured at 9.9 keV. The spatial axes are given in pixels (px). (a) Average hologram of the empty capillary and (b) averaged hologram of the capillary and cell (30 s accumulated exposure time) after empty-beam correction. Shown are the central areas of the holograms without the signal of the capillary walls. Both holograms contain signal from debris (white arrows); the rectangular boxes indicate the regions of interest, and the dashed lines the reconstruction support. (c) Hologram of the cell after alignment, intensity scaling and division of the hologram in (b) by the hologram in (a). (d) Rectangular region of interest from the cell hologram in (c) that is used for phase reconstruction during later stages of the analysis process. Scale bars: 10 μm.

To reconstruct the projections of the cells from the holograms, we create a multi-area support constraint in the plane of reconstruction, which we refer to as reconstruction support and which contains single cells, debris or remaining artifacts from alignment errors. Each hologram is further cropped to a rectangle that contains the cell (see Fig. S1) and reconstructed based on the contrast transfer function (CTF) with a β/δ of $BaSO_4$ (0.0663 at 9.9 keV; 0.0372 at 13.8 keV), a maximum phase shift of 0 rad and the regularization parameters 10^{-3} and 10^{-1} for the low and the high frequencies,

respectively. The resulting CTF reconstruction serves as a phase-guess for the relaxed averaged alternating reflection algorithm (RAAR) (Luke, 2005; Lohse *et al.*, 2020) with 500 iterations that is also constrained by the same reconstruction support and a maximum phase shift of 0 rad. Note that for the CTF reconstruction the use of a support is an optional feature (Huhn *et al.*, 2022) implemented in the Matlab HoloTomoToolbox (Lohse *et al.*, 2020). As the RAAR algorithm requires a reconstruction support, we chose to include it for the CTF reconstruction.

We draw masks to distinguish between debris, $BaSO_4$ grains and the remaining cell body in the reconstructions. To obtain an estimate of the noise level in the holograms a 2D quadratic function $f(y, z)$ is fitted to the data in a moving window with side length 5 pixels. The square root of the variance of the differences between $f(y, z)$ and the value of the hologram at $H_{t,\text{cell},E}(y, z)$ at the same coordinate characterizes the noise level $\sigma_{\text{holo}}(t)$ in the holograms for different accumulated exposure times t and a given energy E.

X-Ray Phase-Contrast Tomography We demonstrate a procedure to obtain a tomographic reconstruction of the $BaSO_4$ grains on the cell surface for a single data set of a fixed and stained cell at 9.9 keV. The analysis is based on (Nicolas *et al.*, 2018) and describes the motion of the trapped cell including a translation and three Euler angles. Therefore, it is able to (i) describe the cellular rotation around multiple rotation axes and (ii) offers the possibility to analyze large data sets with multiple rotations at once. The data sets of the rotating cells are analyzed in analogy to the non-rotating cells except that the background division is not performed on the accumulated but on the individual holograms. In this case, a large single rectangular reconstruction support is drawn that is valid for all projections and is large enough to cover the area of the cell even when it translates during acquisitions. From the CTF reconstruction masks

for the $BaSO_4$ grains are extracted by median-filtering (5×5) and a threshold of -0.115 rad. The center of mass for every grain is manually indexed such that positions in different projections that belong to the same grain exhibit the same label. We characterize every projection by a set of Euler angles and translations: The ith $BaSO_4$ grains on the cell surface in projection n possess the coordinates $\vec{R_i} = (X_i, Y_i, Z_i)$ in the cell-fixed reference frame and is assumed to be static. 3D rotation matrices $M_v(\alpha_n)$ around axis v with angle α_n and a translation $\vec{r_{0,n}} = (0.0, y_{0,n}, z_{0)}$ transform them into the laboratory frame $\vec{r}_{i,n,\text{calc}} = (y_{i,n,\text{calc}}, z_{i,n,\text{calc}})$:

$$\vec{r}_{i,n,\text{calc}} = \Big(M_y(\alpha_n) \cdot M_x(\beta_n) \cdot M_y(\gamma_n) \cdot \vec{R_i} + \vec{r_{0,n}}\Big)_{y,z}. \tag{3}$$

The problem to extract the Euler angles, the translation and $\vec{R_i}$ is formulated as an inverse problem with minimization function $f(n)$ and solved with simulated annealing as implemented in the python function dual_annealing (scipy, v1.9.3) (Xiang *et al.*, 1997). $f(n)$ minimizes the distances between the grain coordinates from the mathematical description (eq. 3) and the experiment and imposes the constraints that the grains are located on the surface of a sphere and that the movement between different projections is small. The former is expressed by the term $Var_i(\vec{R_i})$ in equation (4) below. α_n and γ_n are restricted to $[0, 2\pi]$ and β_n is restricted to $[-\pi/2, \pi/2]$. Moreover, we choose $\alpha_0 = \beta_0 = \gamma_0 = 0$ rad for the first projection. The first 30 images (projections 0 to 29) are used to determine z_0 and $\vec{R_i}$. The obtained values are kept

for all the following projections:

$$
\begin{aligned}
& n \leq 29: \\
& \qquad \min_{\alpha_n,\beta_n,\gamma_n,y_{0,n},z_0,X_i,Y_i,Z_i} \sum_{n=0}^{29} f(n), \\
& n > 29: \\
& \qquad \min_{\alpha_n,\beta_n,\gamma_n,y_{0,n}} f(n), \\
f(n) &= 0.5 \cdot \sum_{i=1}^{3} \left\| \vec{r}_{i,n,\mathrm{exp}} - \vec{r}_{i,n,\mathrm{calc}} \right\|_2^2 + 0.25 \cdot \underbrace{Var_i(\vec{R}_i)}_{\text{marker on surface}} \\
&+ 0.25 \cdot \underbrace{\sum_{i=1}^{3} \begin{cases} 0.0, & n = 0 \\ 0.0, & \left\| \vec{r}_{i,n,\mathrm{calc}} - \vec{r}_{i,n-1,\mathrm{calc}} \right\|_2 < 10 \\ \left(\left\| \vec{r}_{i,n,\mathrm{calc}} - \vec{r}_{i,n-1,\mathrm{calc}} \right\|_2 - 10 \right)^2, & \text{otherwise} \end{cases}}_{\text{small movements}} .
\end{aligned}
\tag{4}
$$

A 3D representation of the grains is obtained by applying the scheme of the simultaneous iterative reconstruction technique (SIRT) (Gordon, 1974; Natterer, 2001; Kak & Slaney, 2001; Salditt *et al.*, 2017). The SIRT algorithm uses non-normalized weights $w_{i,j}$ that determine how much the value of a quantity in voxel v_j is affected by the same quantity projected in pixel p_i, (Kak & Slaney, 2001). The calculation of the weights is more complicated for our rotational description than for the usual tomographic reconstruction, which uses only a single tomographic rotation angle. Therefore, we divide every voxel into m sub-volumes and count how many centers $m_{j,i}$ of sub-volumes in this voxel are located in the ray. Consequently, the weight is given as $w_{i,j} = m_{j,i}/m$. For our reconstruction we compared $m = 1$, $m = 8$, and $m = 64$ sub-volumes per voxel (see Fig. S4d). The projections used in the SIRT algorithm contain only the reconstructed and segmented phase shift signal from the $BaSO_4$ grains. The resulting 3D representation is translated into an electron density $\rho_e(x, y, z)$ (Cloetens *et al.*, 1999):

$$
\rho_e(x, y, z) = \frac{-\phi(x, y, z)}{r_e \cdot \lambda \cdot d}.
$$

Here, $\phi(x, y, z)$ is the phase shift found in a voxel at position (x, y, z) in the reconstruction volume, λ is the wavelength of the X-rays, r_e the the classic electron radius, and d is the side length of a voxel.

2. Reconstructions from XPCI

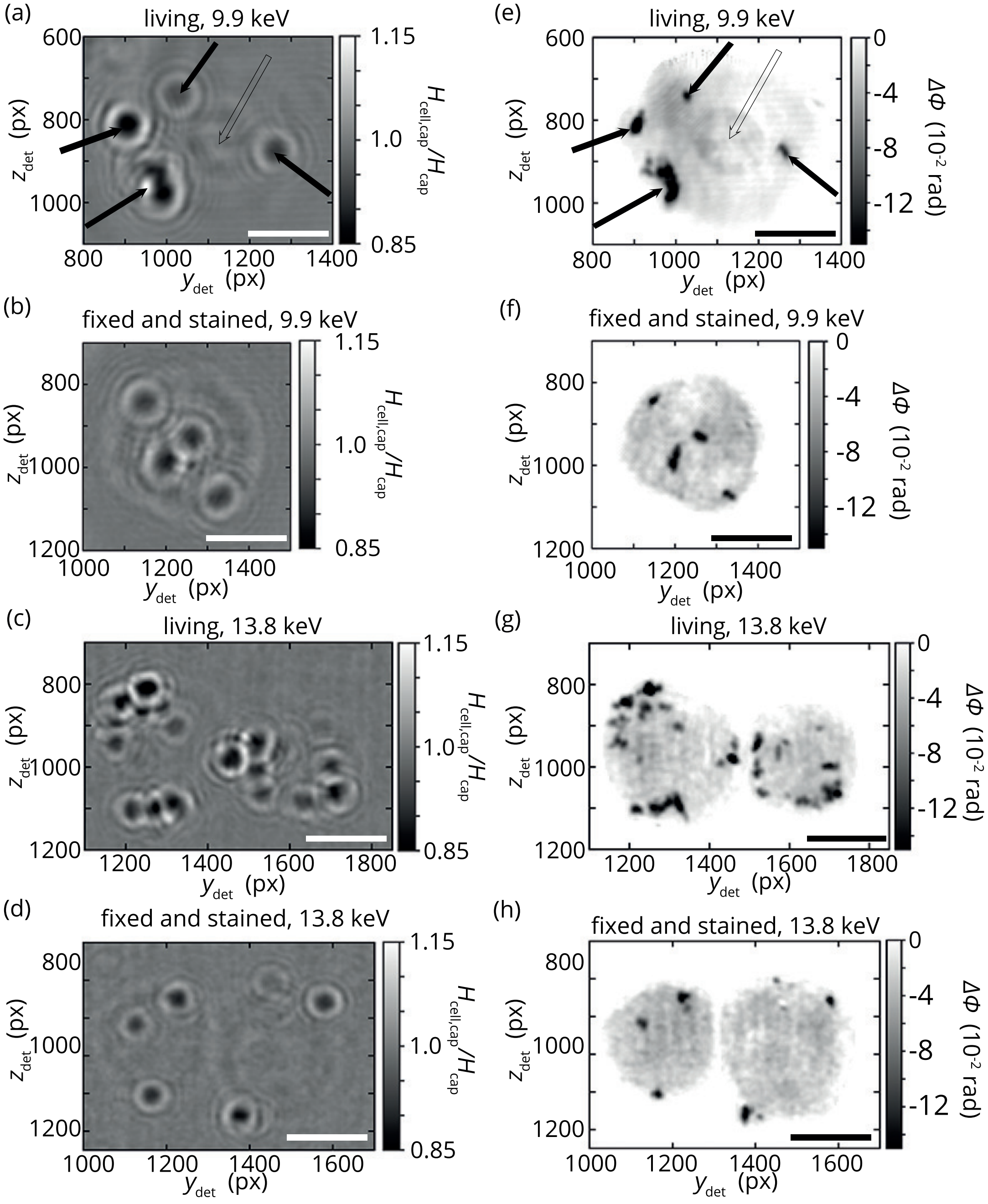

Fig. S2. Figure caption on the following page.

Fig. S2. (a-d) Background-corrected holograms and (e-h) reconstruction of representative living and fixed and stained cells for 9.9 keV and 13.8 keV. In contrast to Fig. 4 in the main text no reconstruction support is drawn. The exposure time for the holograms is 30 s. Black dots in the holograms and reconstructions represent $BaSO_4$-grains (see black filled arrows for examples) that are attached to cells. Scale bars: 10 μm. (e-h) Reconstructed phase shift caused by the holograms in a-d. A CTF reconstruction is used as initial phase guess which is refined using the RAAR algorithm with 500 iterations. (e) The cell nucleus is visible in some reconstructions of living cells that are recorded at 9.9 keV (see black open arrow). The spatial axes are given in pixels (px).

3. Rotation Angles and Translations from XPCT

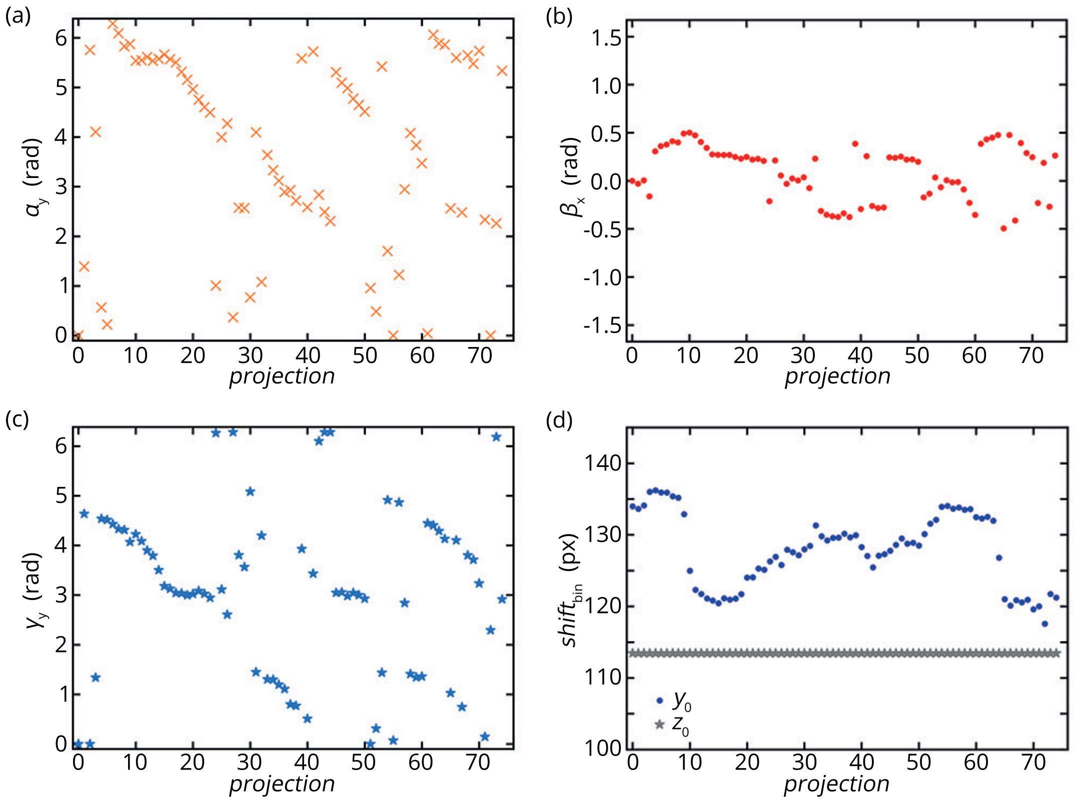

Fig. S3. (a,b,c) Euler angles α_y, β_x and γ_y and (d) translation obtained from the transformation between the reference frame of the cell and the reference frame of the lab as described in section 3.5 in the main text. Shown are the values of the first 75 projections. The spatial axes are given in pixels (px).

4. 3D Reconstruction

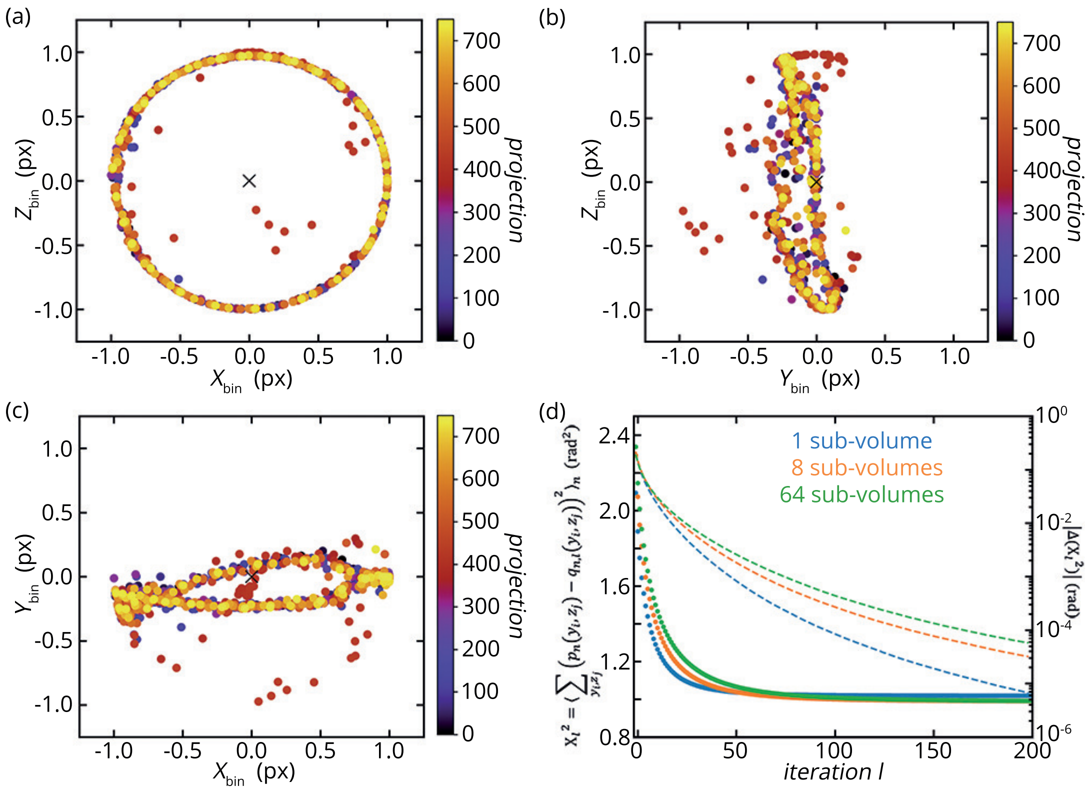

Fig. S4. (a,b,c) Projections of the direction of origin of the X-ray beam in the frame of reference of the cell to the (a) XZ plane, (b) YZ plane, and (c) XY plane from Fig. 5e in the main text. The spatial axes are given in pixels (px). (d) Plot of the average sum of squared differences χ_l^2 between segmented $BaSO_4$ grains $p_n(y_i, z_j)$ in the experimental projections and the forward projection $q_{n,l}(y_i, z_j)$ from the 3D reconstruction (l iterations) (filled circles) and its difference for adjacent projections (dashed lines). $q_{n,l}(y_i, z_j)$ and $p_n(y_i, z_j)$ are phase shifts.

References

Cloetens, P., Ludwig, W., Baruchel, J., Van Dyck, D., Van Landuyt, J., Guigay, J. P. & Schlenker, M. (1999). *Appl. Phys. Lett.* **75**(19), 2912–2914.

Gordon, R. (1974). *IEEE Trans. Nucl. Sci.* **21**(3), 78–93.

Huhn, S., Lohse, L. M., Lucht, J. & Salditt, T. (2022). *Opt. Express,* **30**(18), 32871.

Justusson, B. I. (1981). In *Two-Dimensional Digital Signal Prcessing II*, vol. 43, pp. 161–196. Springer-Verlag.

Kak, A. C. & Slaney, M. (2001). *Principles of computerized tomographic imaging.* SIAM.

Lohse, L. M., Robisch, A.-L., Töpperwien, M., Maretzke, S., Krenkel, M., Hagemann, J. & Salditt, T. (2020). *J. Synchrotron Rad.* **27**(3), 852–859.

Luke, D. R. (2005). *Inverse Probl.* **21**(1), 37–50.

Natterer, F. (2001). *The mathematics of computerized tomography.* SIAM.

Nicolas, J.-D., Hagemann, J., Sprung, M. & Salditt, T. (2018). *J. Synchrotron Rad.* **25**(4), 1196–1205.

Osterhoff, M. (2017). *J. Phys.: Conf. Ser.* **849**, 012059.

Salditt, T., Aspelmeier, T. & Aeffner, S. (2017). *Biomedical imaging: principles of radiography, tomography and medical physics.* Walter de Gruyter GmbH & Co KG.

TheHDFGroup, (1997). Hierarchical Data Format.

Van Rossum, G. & Drake, F. L. (2009). *Python 3 Reference Manual.* CreateSpace.

Xiang, Y., Sun, D., Fan, W. & Gong, X. (1997). *Phys. Lett. A,* **233**(3), 216–220.
